# Lithium Reduces Migration and Collagen Synthesis Activity in Human Cardiac Fibroblasts by Inhibiting Store-Operated Ca^2+^ Entry

**DOI:** 10.3390/ijms22020842

**Published:** 2021-01-15

**Authors:** Pao-Huan Chen, Cheng-Chih Chung, Yuan-Feng Lin, Yu-Hsun Kao, Yi-Jen Chen

**Affiliations:** 1 Graduate Institute of Clinical Medicine, College of Medicine, Taipei Medical University, Taipei 11031, Taiwan; b8601115@tmu.edu.tw (P.-H.C.); d001089012@tmu.edu.tw (Y.-F.L.); 2 Department of Psychiatry, Taipei Medical University Hospital, Taipei 11031, Taiwan; 3 Department of Psychiatry, School of Medicine, College of Medicine, Taipei Medical University, Taipei 11031, Taiwan; 4 Division of Cardiovascular Medicine, Department of Internal Medicine, Wan Fang Hospital, Taipei Medical University, Taipei 11696, Taiwan; michaelchung110@gmail.com; 5 Division of Cardiology, Department of Internal Medicine, School of Medicine, College of Medicine, Taipei Medical University, Taipei 11031, Taiwan; 6 Cell Physiology and Molecular Image Research Center, Wan Fang Hospital, Taipei Medical University, Taipei 11696, Taiwan; 7 Department of Medical Education and Research, Wan Fang Hospital, Taipei Medical University, Taipei 11696, Taiwan; 8 Cardiovascular Research Center, Wan Fan Hospital, Taipei Medical University, Taipei 11696, Taiwan

**Keywords:** lithium, antifibrosis, cardioprotection, Ca^2+^ release-activate Ca^2+^ channel, intracellular Ca^2+^ homeostasis

## Abstract

Cardiac fibrosis plays a vital role in the pathogenesis of heart failure. Fibroblast activity is enhanced by increases in store-operated Ca^2+^ entry (SOCE) and calcium release-activated calcium channel protein 1 (Orai1) levels. Lithium regulates SOCE; however, whether therapeutic concentrations of lithium can be used to inhibit cardiac fibrogenesis is unknown. Migration and proliferation assays, Western blotting, real-time reverse-transcription polymerase chain reaction analysis, and calcium fluorescence imaging were performed in human cardiac fibroblasts treated with or without LiCl at 1.0 mM (i.e., therapeutic peak level) or 0.1 mM (i.e., therapeutic trough level) for 24 h. Results showed that LiCl (0.1 mM, but not 1.0 mM) inhibited the migration and collagen synthesis ability of cardiac fibroblasts. Additionally, thapsigargin-induced SOCE was reduced in fibroblasts treated with LiCl (0.1 mM). The expression level of Orai1 was lower in LiCl (0.1 mM)-treated fibroblasts relative to the fibroblasts without LiCl treatment. Fibroblasts treated with a combination of LiCl (0.1 mM) and 2-APB (10 μM, an Orai1 inhibitor) demonstrated similar migration and collagen synthesis abilities as those in LiCl (0.1 mM)-treated fibroblasts. Altogether, lithium at therapeutic trough levels reduced the migration and collagen synthesis abilities of human cardiac fibroblasts by inhibiting SOCE and Orai1 expression.

## 1. Introduction

Bipolar disorder is a chronic recurrent disease characterized by episodic mood symptoms, cognitive deficits, and high burden of medical comorbidities during illness progression [1,2,3]. Accumulating evidence has indicated that patients with bipolar disorder have a 2–4-times higher mortality rate and at least a 10-year reduction in life expectancy compared to those in the general population [3,4]. Among the causes of natural deaths, studies from both Eastern and Western countries have consistently shown that cardiac disease is among the leading ones, resulting in the excess and premature deaths of patients with bipolar disorder [4,5].

Cardiac fibrosis is a detrimental factor in a variety of heart diseases [6,7]. The excessive accumulation of extracellular matrix proteins interferes with electrical conduction, causes myocardial stiffness, contributes to cardiac arrhythmias, and increases the rate of heart failures [7]. Consistent with the literature [8], our recent studies have demonstrated that Ca^2+^ entry is a crucial signal that regulates the pro-fibrotic activity of human cardiac fibroblasts [9,10]. Among the various types of Ca^2+^ entry through ion channels in the plasma membrane, the dysfunction of store-operated Ca^2+^ entry has recently been found to be the key contributor to heart failure [11,12,13,14]. Cardiac fibroblasts isolated from patients with heart failure have been reported to possess an enhanced ability to synthesize collagen, which is related to the increase in Ca^2+^ entry via elevation in store-operated Ca^2+^ entry and the expression level of calcium release-activated calcium channel protein 1 (Orai1) [13].

Lithium has been recommended as the first-line treatment for mood symptoms in patients with bipolar disorder by most treatment guidelines [15]. In addition to having mood-stabilizing effects, many large-scale epidemiological studies have demonstrated that lithium in drinking water reduces the incidence of dementia and risk of atherosclerotic heart death, and promotes longevity [16,17,18]. Moreover, emerging evidence has indicated a decrease in the risk of cardiovascular morbidity and mortality in patients with bipolar disorder after receiving therapeutic lithium [19,20]. These findings suggest that lithium may have potential protective effects on the heart [21]. Laboratory studies have demonstrated that lithium can regulate store-operated Ca^2+^ entry in cortical neurons, lymphocytes, and fibroblasts [22,23,24]. While lithium increases store-operated Ca^2+^ entry at concentrations above the therapeutic peak level (2 mM) [22,23], lithium inhibits such entry at concentrations within the therapeutic range (0.75 mM) [24]. The mechanisms for these modulating effects may involve the regulation of Orai1 expression [23].

The aim of this study was to examine whether therapeutic peak (1.0 mM) or trough levels (0.1 mM) of lithium [25,26] inhibit the pro-fibrotic activity of human cardiac fibroblasts through the modulation of store-operated Ca^2+^ entry.

## 2. Results

### 2.1. Effects of Therapeutic Trough Levels of Lithium on Cardiac Fibroblasts

**Figure 1:**
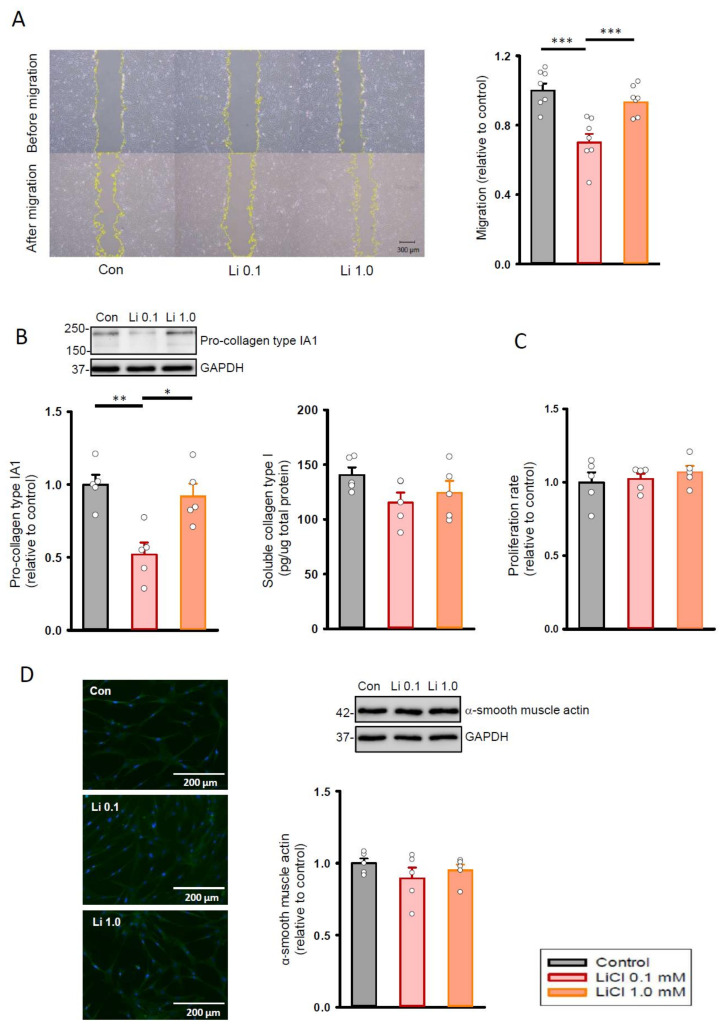
Cell migration, proliferation, and collagen synthesis abilities of human cardiac fibroblasts with and without LiCl treatment for 24 h. (**A**) Photograph and graph presenting mean data of the migration assay in human cardiac fibroblasts (*n* = 7 from four independent experiments). (**B**) Photograph and bar graph presenting average data of the pro-collagen type IA1 (*n* = 5 from three independent experiments) and soluble collagen type I (*n* = 5 from three independent experiments) production in human cardiac fibroblasts. (**C**) Average data of the proliferation rate in the cardiac fibroblasts (*n* = 5 from three independent experiments). (**D**) Photograph of immunofluorescence microscopy imaging and graph presenting average data of the α-smooth muscle actin expression on Western blot (*n* = 5 from three independent experiments). GAPDH was used as a loading control. One-way repeated measures analysis of variance (ANOVA) test with Tukey’s post hoc test was used to compare the human cardiac fibroblasts under different treatment conditions. * *p* < 0.05, ** *p* < 0.01, *** *p* < 0.001.

As shown in Figure 1A, lithium (0.1 mM)-treated cardiac fibroblasts exhibited a lower migration ability when compared with the control and the lithium (1.0 mM)-treated cardiac fibroblasts. In addition, lithium (0.1 mM)-treated cardiac fibroblasts had a lower production of pro-collagen type IA1 than the control (Figure 1B). However, the control and lithium (0.1 mM)-treated fibroblasts did not have a significant difference in soluble collagen type I amount. In addition, lithium (0.1 mM)-treated fibroblasts had similar proliferation rate (Figure 1C), expression of α-smooth muscle actin (Figure 1D), and cell size (Supplementary Figure S1) as compared to the control and lithium (1.0 mM)-treated human cardiac fibroblasts.

**Figure 2:**
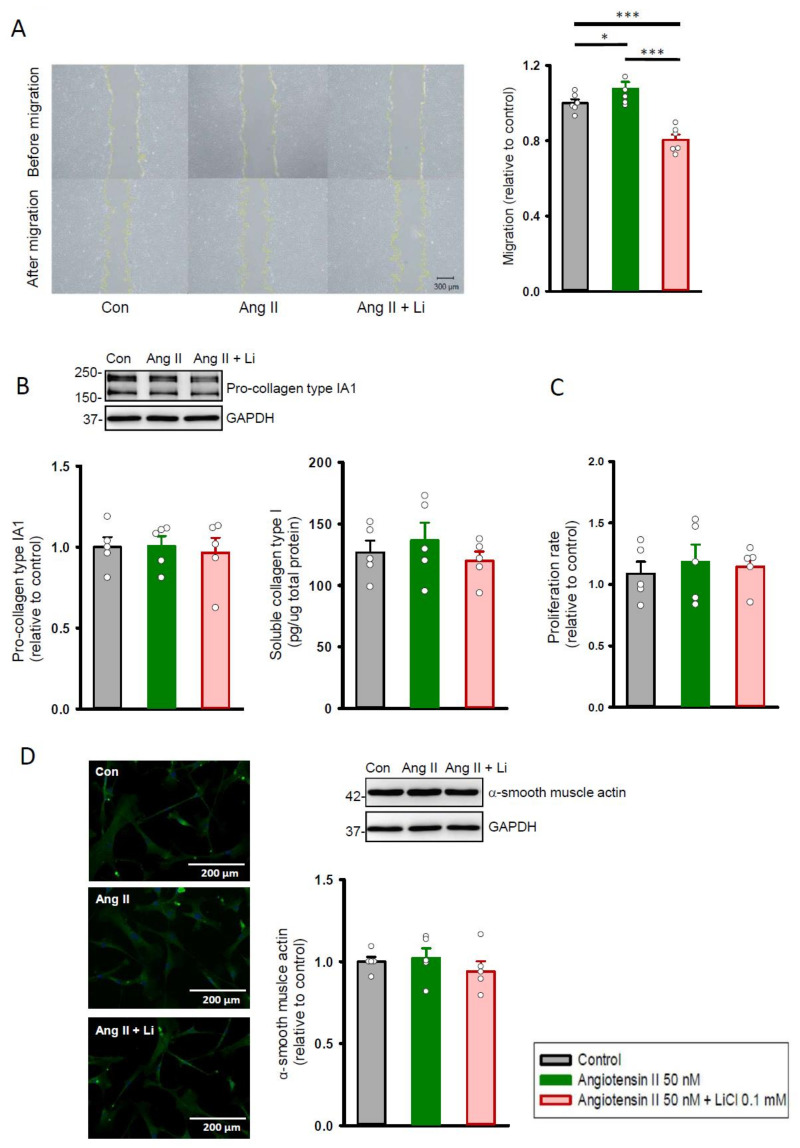
Cell migration, proliferation, and collagen synthesis abilities of human cardiac fibroblasts with and without LiCl/angiotensin II treatment for 24 h. (**A**) Photograph and graph presenting mean data of the migration assay in human cardiac fibroblasts (*n* = 6 from three independent experiments). (**B**) Photograph and bar graph presenting average data of the pro-collagen type IA1 (*n* = 5 from three independent experiments) and soluble collagen type I (*n* = 5 from three independent experiments) production in human cardiac fibroblasts. (**C**) Average data of the proliferation rate in the cardiac fibroblasts (*n* = 5 from three independent experiments). (**D**) Photograph of immunofluorescence microscopy imaging and graph presenting average data of the α-smooth muscle actin expression on Western blot (*n* = 5 from three independent experiments). GAPDH was used as a loading control. One-way repeated measures analysis of variance (ANOVA) test with Tukey’s post hoc test was used to compare the human cardiac fibroblasts under different treatment conditions. * *p* < 0.05, *** *p* < 0.001.

After stimulation with angiotensin II (50 nM), cardiac fibroblasts had a greater migration ability than the control (Figure 2A). A combination of angiotensin II (50 nM) and lithium (0.1 mM) reduced the migration ability of cardiac fibroblasts as compared to the control and angiotensin II (50 nM)-stimulated cells. There was no significant difference observed in the production of pro-collagen type IA1 (Figure 2B), proliferation rate (Figure 2C), and expression of α-smooth muscle actin (Figure 2D) between the control, angiotensin II (50 nM) alone, and combined angiotensin II (50 nM) and lithium (0.1 mM)-treated cells.

### 2.2. Therapeutic Trough Levels of Lithium Reduce Store-Operated Ca^2+^ Entry

**Figure 3:**
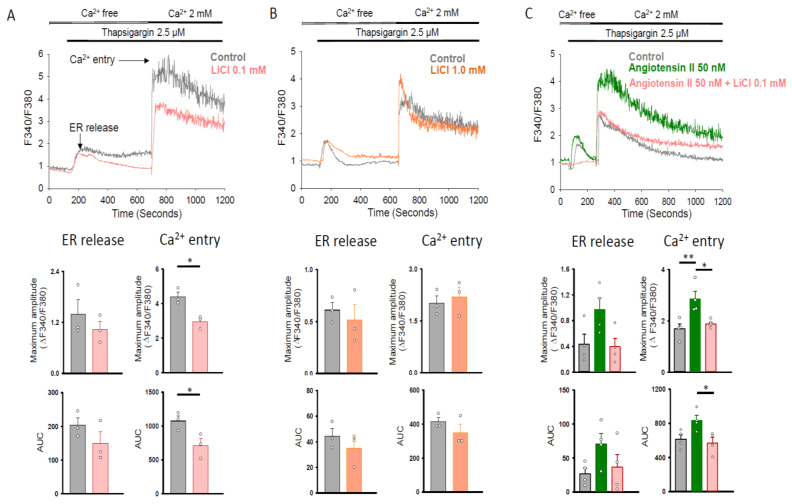
Store-operated Ca^2+^ entry in human cardiac fibroblasts with and without LiCl/angiotensin II treatment for 24 h. Representative tracings and average data of the maximum amplitude and AUC of the intracellular Ca^2+^ concentration in Fura-2 AM-loaded human cardiac fibroblasts following the removal of extracellular Ca^2+^, the addition of the sarco/ER Ca^2+^-ATPase inhibitor thapsigargin (2.5 μM), and the re-addition of extracellular Ca^2+^ (2 mM). Panel (**A**) shows that LiCl (0.1 mM)-treated cells had a similar maximum amplitude and AUC of ER release and a lower maximum amplitude and AUC of Ca^2+^ entry than the control cells (*n* = 3 independent experiments). Panel (**B**) shows that LiCl (1.0 mM) and control cells had a similar maximum amplitude and AUC of ER release and Ca^2+^ entry (*n* = 3 independent experiments). Panel (**C**) shows that angiotensin II (50 nM)-treated cells had a larger maximum amplitude of Ca^2+^ entry than the control cells. Combined lithium (0.1 mM) and angiotensin II (50 nM)-treated cells had a smaller maximum amplitude and AUC of Ca^2+^ entry compared to the angiotensin II (50 nM)-treated cells (*n* = 4 independent experiments). Unpaired *t* test, Mann–Whitney rank-sum test, and one-way analysis of variance (ANOVA) test with Tukey’s post hoc test were used to compare the ER release and Ca^2+^ entry under different treatment conditions. * *p* < 0.05, ** *p* < 0.01.

Figure 3 illustrates the tracings of the intracellular Ca^2+^ concentration in Fura-2 AM-loaded human cardiac fibroblasts following the removal of extracellular Ca^2+^, the addition of thapsigargin (2.5 μM; a sarco/endoplasmic reticulum [ER] Ca^2+^-ATPase inhibitor), and the re-addition of extracellular Ca^2+^ (2 mM) in fibroblasts with and without lithium (0.1 and 1.0 mM) treatment. We noted no significant difference in the maximum amplitude or integrated Ca^2+^ signal (area under curve, AUC) of ER release between the control and lithium (0.1 and 1.0 mM)-treated fibroblasts (Figure 3A,B). In addition, the lithium (0.1 mM)-treated fibroblasts had a lower maximum amplitude and AUC of Ca^2+^ entry as compared with the control (Figure 3A). In contrast, the control and lithium (1.0 mM)-treated human cardiac fibroblasts had a similar maximum amplitude and AUC of Ca^2+^ entry (Figure 3B). As shown in Figure 3C, angiotensin II 50 (nM)-treated human cardiac fibroblasts had a higher maximum amplitude of Ca^2+^ entry than that of the control cells. In the presence of angiotensin II (50 nM), lithium (0.1 mM) also reduced the maximum amplitude and AUC of Ca^2+^ entry in human cardiac fibroblasts.

### 2.3. Therapeutic Trough Levels of Lithium Decrease Orai1 Expression

**Figure 4:**
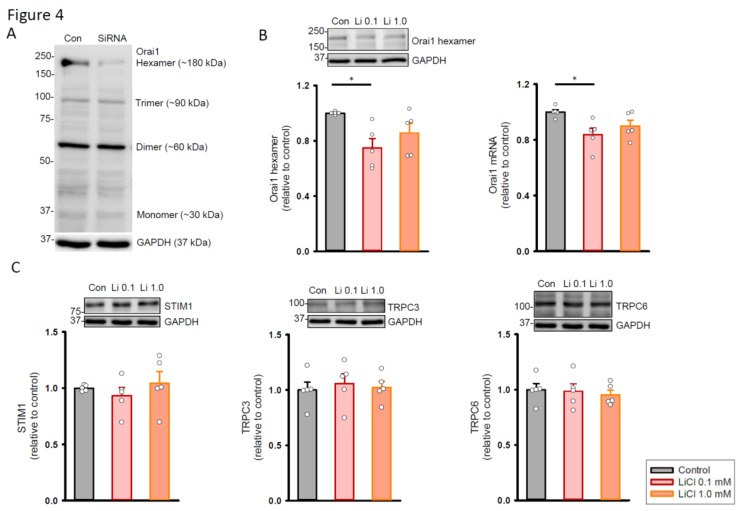
Expression of store-operated Ca^2+^ channel proteins in human cardiac fibroblasts with and without LiCl treatment for 24 h. (**A**) Photograph showing the Western blot of Orai1 in human cardiac fibroblasts with and without siRNA treatment for 48 h. (**B**) Photograph and graph showing the mean data of Orai1 protein (*n* = 5 from four independent experiments) and mRNA (*n* = 5 from three independent experiments) expression in human cardiac fibroblasts. GAPDH was used as a loading control. (**C**) Photograph and graph showing the mean data of STIM1, TRPC3, and TRPC6 protein expression in human cardiac fibroblasts (*n* = 5 from three independent experiments). GAPDH was used as a loading control. One-way repeated measures analysis of variance (ANOVA) test with Tukey’s post hoc test was used to compare the expression levels of proteins or mRNAs under different treatment conditions. * *p* < 0.05.

Because Orai1 is a plasma membrane pore-forming channel that functions in the hexameric structure, human cardiac fibroblasts were transfected with small interfering (si)RNAs against the Orai1 gene for 48 h to determine the specificity of the antibody staining in the Western blot (Figure 4A). The full blot showed that the expression levels of Orai1 hexamers were decreased in 68.5% after siRNA knockdown. Accordingly, we compared the expression levels of Orai1 hexamers between the control and lithium (0.1 and 1.0 mM)-treated cells. As shown in Figure 4B, the lithium (0.1 mM)-treated fibroblasts had lower expression levels of Orai1 hexamers and messenger (m)RNAs compared to the control. On the other hand, the control and lithium (1.0 mM)-treated human cardiac fibroblasts had similar expression levels of Orai1 hexamers and mRNAs. Given that stromal interaction molecule 1 (STIM1) activates Orai1 upon depletion of ER Ca^2+^ in cardiac fibroblasts and transient receptor potential canonical (TRPC) channels are the important ion channels that regulate Ca^2+^ entry in cardiac fibrosis, this study also investigated the effects of lithium on the expression of STIM1, TRPC3, and TRPC6 proteins. The expression levels of STIM1, TRPC3, and TRPC6 proteins were similar between the lithium (0.1 and 1.0 mM)-treated fibroblasts and control cells (Figure 4C).

### 2.4. Orai1 Inhibitor 2-APB on the Effects of Lithium in Cardiac Fibroblasts

**Figure 5:**
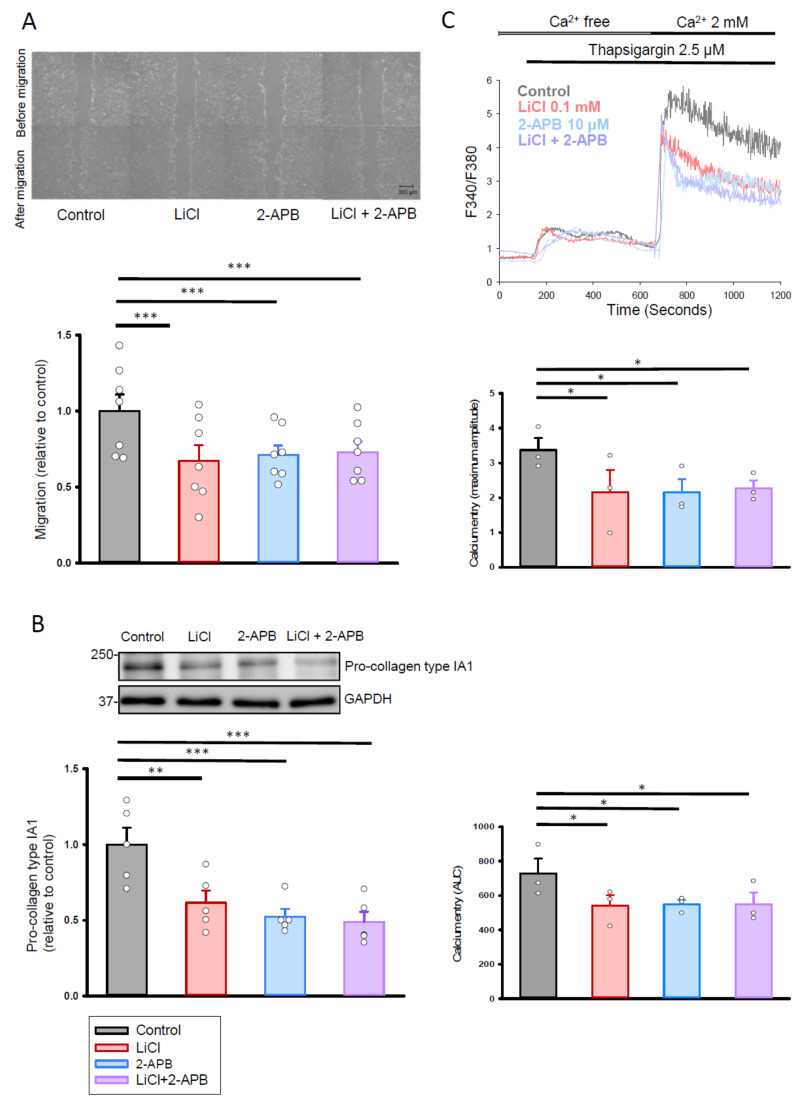
Cell migration, collagen synthesis ability, and store-operated Ca^2+^ entry in human cardiac fibroblasts treated with LiCl (0.1 mM) and 2-APB (10 μM) for 24 h. (**A**) Photographs and average data of the migration assay in human cardiac fibroblasts (*n* = 7 from four independent experiments). (**B**) Photographs and average data of the pro-collagen type IA1 production (*n* = 5 from three independent experiments) in human cardiac fibroblasts. (**C**) Representative tracings and average data of the maximum amplitude and AUC of intracellular Ca^2+^ concentration following the re-addition of extracellular Ca^2+^ (2 mM) in control, LiCl (0.1 mM), 2-APB (10 μM), or combined lithium (0.1 mM) and 2-APB (10 μM)-treated human cardiac fibroblasts (*n* = 3 independent experiments). One-way repeated measures analysis of variance (ANOVA) test with Tukey’s post hoc test was used to compare the human cardiac fibroblasts under different treatment conditions. * *p* < 0.05, ** *p* < 0.01, *** *p* < 0.001.

To examine whether the effects of lithium at the therapeutic trough level on cardiac fibroblasts were blocked following Orai1 inhibition, the Orai1 inhibitor 2-aminoethoxydiphenyl borate (2-APB) was added to the human cardiac fibroblasts with or without lithium (0.1 mM) treatment. Treatment with lithium (0.1 mM) alone, 2-APB (10 μM) alone, and the combination of lithium (0.1 mM) and 2-APB (10 μM) reduced the migration ability of the human cardiac fibroblasts when compared with the control (Figure 5A). In addition, the combination of 2-APB (10 μM) and lithium (0.1 mM) treatment inhibited the migration ability of human cardiac fibroblasts to a similar extent as that observed in the lithium (0.1 mM)-treated fibroblasts. Similarly, combined lithium (0.1 mM) and 2-APB (10 μM)-treated fibroblasts exhibited comparable reductions in the production of pro-collagen type IA1 (Figure 5B) and Ca^2+^ entry (Figure 5C) as compared with the lithium (0.1 mM)-treated cells.

### 2.5. Therapeutic Trough Levels of Lithium Increase Phosphorylated Akt Expression

**Figure 6:**
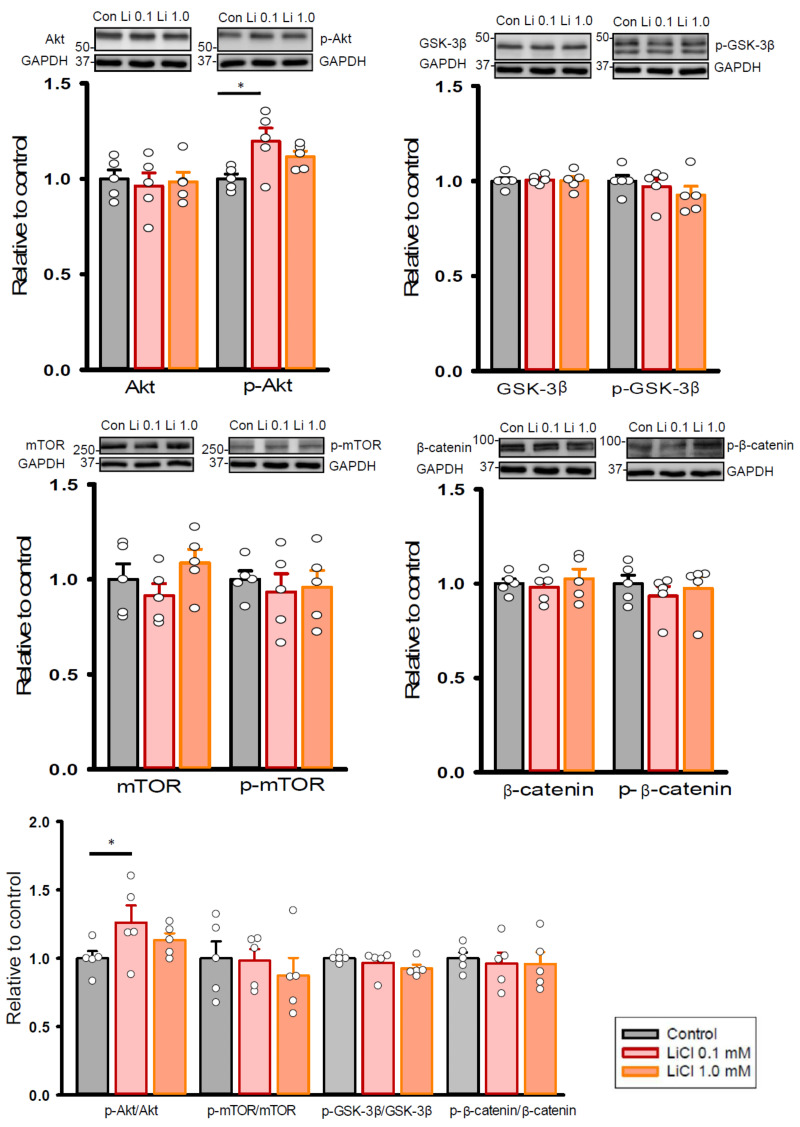
Expression levels of pro-fibrotic signaling proteins in human cardiac fibroblasts with and without LiCl treatment for 24 h. Photograph and graph showing the mean data of expression levels and ratios of Akt, mTOR, GSK-3β, and β-catenin in human cardiac fibroblasts (*n* = 5 from three independent experiments). GAPDH was used as a loading control. One-way repeated measures analysis of variance (ANOVA) test with Tukey’s post hoc test was used to compare the expression levels of proteins under different treatment conditions. * *p* < 0.05.

Considering that pro-fibrotic signaling involves Akt/mammalian target of rapamycin (mTOR) or glycogen synthase kinase 3 beta (GSK-3β)/β-catenin cascade, we examined whether therapeutic trough levels of lithium alert these pathways in human cardiac fibroblasts. As shown in Figure 6, lithium (0.1 mM)-treated fibroblasts exhibited increased expression levels of phosphorylated Akt and ratios of phosphorylated Akt to total Akt levels as compared to the control cells. On the other hand, control and lithium (0.1 and 1.0 mM)-treated fibroblasts had similar expression levels of total and phosphorylated mTOR, GSK-3β, and β-catenin.

## 3. Discussion

To our knowledge, this is the first study to explore the modulating effects of therapeutic lithium on the pro-fibrotic activity of human cardiac fibroblasts with respect to store-operated Ca^2+^ entry. At therapeutic trough levels (0.1 mM), lithium reduced the migration and collagen synthesis abilities of human cardiac fibroblasts through its inhibitory actions on store-operated Ca^2+^ entry and Orai1 expression. Furthermore, at therapeutic peak levels (1.0 mM), lithium did not enhance the pro-fibrotic capability of human cardiac fibroblasts. Consistent with previous study findings that lithium above therapeutic peak levels (>1.0 mM) increases the pro-fibrotic abilities of fibroblasts [22,27], the findings of the present study suggest that the effects of lithium on the pro-fibrotic activity in cardiac fibroblasts may be in a dose-dependent manner.

Our findings of an association between a reduction in store-operated Ca^2+^ entry and the inhibitory effects of lithium on migration and collagen synthesis in human cardiac fibroblasts may provide a novel mechanism that explains the observed cardioprotective effects of lithium across several epidemiological and clinical studies. In addition, the inhibitory effects of therapeutic lithium on cardiac fibroblasts were related to the downregulation of Orai1 expression. This finding is consistent with those of prior studies, which showed the protective effect of Orai1 inhibition against fibrosis [28,29]. In particular, several studies have demonstrated that receptor-operated Ca^2+^ entry mediated by TRPC channels is an important ion channel that regulates Ca^2+^ entry in cardiac fibroblasts [12,30,31]. Furthermore, therapeutic lithium suppresses Ca^2+^ entry by downregulating TRPC3 in cells such as astrocytes and lymphocytes [32,33]. However, little is known in the literature regarding the regulation of TRPC channels in cardiac fibroblasts by lithium. In the present study, we did not find a modulating effect of therapeutic lithium on TRPC3 and TRPC6 expression in human cardiac fibroblasts. Our findings suggest that the inhibitory effect of therapeutic lithium on store-operated Ca^2+^ entry in cardiac fibroblasts is mediated by Orai1 rather than TRPC channels.

In a previous animal study, lithium treatment ameliorated pathological hypertrophy and cardiac fibrosis at plasma levels of 0.39 ± 0.06 mM during remodeling [34]. In addition, magnetic resonance spectroscopic studies have shown that the concentrations of lithium in muscular tissues are generally lower than half of the serum concentrations [35]. Accordingly, the lithium level (0.1 mM) used in the current in vitro study is therapeutically close to the plasma level (0.39 mM) used in the aforementioned in vivo study. In the previous animal study [34], the Akt/mTOR-dependent pathway was suggested to enhance physiological hypertrophy with therapeutic lithium. However, the role of the Akt/mTOR-dependent pathway in the effects of lithium on cardiac fibrosis has not been elucidated. In the present in vitro study, the therapeutic trough levels (0.1 mM) of lithium increased the expression levels of phosphorylated Akt in cardiac fibroblasts. This finding contradicts those in the literature, which indicates that the activation of Akt promotes fibrogenesis [36]. However, we did not find significant difference in the expression levels of target proteins regulated by phosphorylated Akt, such as GSK-3β and mTOR, between the lithium (0.1 mM)-treated fibroblasts and control cells. The findings lead us to speculate that the downstream targets of Akt activation for the effects of lithium on cardiac fibroblasts may involve other molecules. Little is known about the effect of Akt activation on the regulation of Orai1 in cardiac fibroblasts. Considering the state of the literature and the present in vitro results, additional in vivo studies are required to confirm the role of therapeutic lithium in reducing cardiac fibrosis by modulating Akt signaling related to Orai1 expression and activity.

GSK-3β is among one of the well-known molecular targets involved in the therapeutic actions of lithium and plays a central role in the regulation of the Wnt/β-catenin signaling pathway [37]. Several publications have indicated a crucial role of Wnt signaling in cardiac fibrogenesis [36]. One previous study has demonstrated that immortalized rat cardiac fibroblasts exhibit increased migration and differentiation abilities regulated by the Wnt signaling [38]. In addition, Wnt signaling promotes the activation, migration, and proliferation of human cardiac fibroblasts treated with transforming growth factor beta 1 (TGF-β1) [39]. However, we did not observe a significant difference in the expression levels of total and the phosphorylated form of GSK-3β as well as its downstream target β-catenin between the lithium-treated fibroblasts and control cells. The findings suggest that the inhibitory effects of lithium (0.1 mM) on migration and collagen synthesis in cardiac fibroblasts may not be mediated by GSK-3β. Compared with previous studies that have utilized a model of TGF-β1 stimulation or immortalized cells [38,39], human cardiac fibroblasts used in our study were isolated from normal adult cardiac tissue. The difference in the experimental models may also explain the discrepancy.

Previous studies have shown that angiotensin II has pro-fibrotic effects on human cardiac fibroblasts [40]. In addition, the expression and localization of Orai1 proteins are strongly regulated by angiotensin II [41,42]. In this study, we found that angiotensin II-stimulated human cardiac fibroblasts possessed a greater migration ability than the control cells. In the presence of lithium (0.1 mM), the migration ability of cardiac fibroblasts was decreased. Furthermore, angiotensin II elevated store-operated Ca^2+^ entry in cardiac fibroblasts. Lithium (0.1 mM) reduced store-operated Ca^2+^ entry in cardiac fibroblasts treated with angiotensin II. These findings suggest that lithium (0.1 mM) may attenuate the effects of angiotensin II (50 nM) in human cardiac fibroblasts. However, we did not observe greater pro-collagen type IA1 expression, proliferation, and myofibroblast conversion in human cardiac fibroblasts treated with angiotensin II for 24 h. These findings were similar to those in previous studies that stimulation with angiotensin II for 24–48 h had no direct effect on collagen type I expression and proliferation ability in human cardiac fibroblasts [40,43,44]. Rather, angiotensin II may indirectly enhance the collagen synthesis and proliferation ability of human cardiac fibroblasts through the activation of autocrine or paracrine signaling, such as TGF-β1 [43,45]. Although the concentration of angiotensin II (50 nM) selected in this study was considerably higher than that measured in humans [46], this study found that angiotensin II (50 nM) has no effect on the most critical characteristics of a pro-fibrotic fibroblast phenotype (myofibroblast conversion and collagen release) in human cardiac fibroblasts. Accordingly, we still do not know whether lithium opposes these pro-fibrotic abilities in human cardiac fibroblasts.

In our study, the amount of soluble collagen type I in cell culture medium did not significantly differ between the control and lithium (0.1 or 1.0 mM) treatment groups, even though the production of pro-collagen type IA1 was reduced in lithium (0.1 mM)-treated cardiac fibroblasts as compared to the control cells. In human cardiac fibroblasts, pro-collagen type I is synthesized from three pro-α collagen chains in the ER [47]. After secretion into the extracellular matrix, the C- and N-terminal pro-peptides of pro-collagen type I are cleaved off by proteinase, which results in the formation of collagen type I [47]. Therefore, our findings suggest that therapeutic trough levels of lithium do not have inhibitory effects on the proteinases involved in the formation of collagen type I from pro-collagen type I. Additionally, it is also possible that fibroblasts deposit a minimal amount of collagen into the culture medium due to the restricted activity of pro-collagen C-proteinase under the culture condition [48,49].

The clinical relevance of our findings is that it provides laboratory evidence for determining the optimal lithium dose in the treatment of bipolar disorder and cardiovascular comorbidities. Most current treatment guidelines recommend for the dose of therapeutic lithium to be based on its mood-stabilizing efficacy [15]. The recommended plasma concentration generally ranges from 0.4 to 1.0 mM. Magnetic resonance spectroscopic studies have previously shown that the corresponding concentrations of lithium in muscular tissue are generally lower than half of the serum concentrations [35]. Therefore, the concentration (0.1 mM) used in the present study was assumed to correlate with the therapeutic trough values of lithium in cardiac tissue. In this in vitro study, the therapeutic trough levels (0.1 mM) of lithium reduced migration and collagen synthesis in human cardiac fibroblasts, and the therapeutic peak levels (1.0 mM) of lithium did not change it. Thus, these findings suggest that the once-daily administration of lithium and the maintenance of the peak and trough levels between 1.0 and 0.1 mM may be useful in reducing cardiovascular morbidity and mortality in patients with bipolar disorder. However, considering the limitations inherent to in vitro studies and the fact that our human cardiac fibroblasts were isolated from normal adult cardiac tissues, future clinical and translational research studies are mandatory to investigate this hypothesis in patients with bipolar disorder.

In conclusion, the results of this study suggest that, at therapeutic trough levels, lithium reduces the migration and collagen synthesis ability of human cardiac fibroblasts through its inhibitory action on store-operated Ca^2+^ entry and Orai1 expression. Future research studies are mandatory to apply pro-fibrotic stimulus to investigate lithium’s effects on the multifaceted activities of human cardiac fibroblasts.

## 4. Materials and Methods

### 4.1. Cell Culture

Human cardiac fibroblasts isolated from normal adult heart tissue (#CC-2903, Lonza, Walkersville, MD, USA) were cultured in an FBM kit (#CC-3131, Lonza, Walkersville, MD, USA) at 37 °C with 5% CO_2_. To avoid pseudo-replication, at least two sets of the cells from passages 4 to 6 were used for each experiment and at least three independent experiments were conducted.

### 4.2. Cell Migration Assay

The migration ability of human cardiac fibroblasts with or without LiCl treatment (#62476, Sigma-Aldrich, St. Louis, MO, USA) was assessed. LiCl was administered at 1.0 mM, the therapeutic peak level [25,26], or 0.1 mM, the therapeutic trough level [25,26], for 24 h. For the experiment in the human cardiac fibroblasts stimulated with a pro-fibrotic stimulus, angiotensin II (50 nM; #A9525, Sigma-Aldrich, St. Louis, MO, USA) was administrated to human cardiac fibroblasts with or without LiCl treatment for 24 h. To examine whether the inhibition of Orai1 can block the effects of lithium at the therapeutic trough level on cardiac fibroblasts, the Orai1 inhibitor, 2-APB (#D9754, Sigma-Aldrich, St. Louis, MO, USA), was added to human cardiac fibroblasts with or without LiCl (0.1 mM) treatment for 24 h.

Before treatment, human cardiac fibroblasts were plated in a 6-well culture dish at a density of 10^5^ cells per well. After growing to nearly full confluence, the cells were incubated in serum-free medium with or without LiCl treatment (1.0 or 0.1 mM) for a total of 24 h. A wound-healing assay was conducted 6 h after a cell monolayer was scraped with a P200 pipette tip. The net migration area was subtracted from that at the baseline as in our previously described methods [9]. For each treatment condition, the average of four individual regions was calculated.

### 4.3. Cell Proliferation Assay

The proliferation of the human cardiac fibroblasts was measured using a commercial MTS kit (#G3582, Promega, Madison, WI, USA) as in our previously described methods [9]. Briefly, human cardiac fibroblasts were plated in a 96-well culture dish at a density of 10^4^ cells per well. After growing to 50% confluence, the cells were incubated in serum-free medium with or without LiCl treatment (1.0 or 0.1 mM)/angiotensin II (50 nM) for 24 h. Cell growth was analyzed using an MTS reagent, which was added 4 h before the spectrophotometric analysis.

### 4.4. Western Blotting

Human cardiac fibroblasts were lysed in protein extraction reagent (#78501, Thermo Fisher Scientific, Loughborough, UK) as in our previously described methods [9], or using the RIPA Lysis and Extraction Buffer (#899901, Thermo Fisher Scientific, Loughborough, UK) for Orai1 experiments. Proteins of equal amounts were resolved by sodium dodecyl sulfate polyacrylamide gel electrophoresis (SDS-PAGE). The proteins were then electrophoretically transferred onto nitrocellulose membranes. Protein concentration was determined using a Qubit protein assay kit (#Q33212, Thermo Fisher Scientific, Loughborough, UK) or Bio-Rad Protein Assay (#500-0006, Bio-Rad Laboratories, CA, USA) for Orai1 protein. Blots were probed with primary antibodies against pro-collagen type IA1 (1:500, monoclonal, #sc-293182, Santa-Cruz Biotechnology, Santa Cruz, CA, USA), α-smooth muscle actin (1:5000, monoclonal, #ab7818, Abcam, Cambridge, UK), Orai1 (1:1000, polyclonal, #4281, ProSci Incorporated, Poway, CA, USA), STIM1 (1:1000, monoclonal, #610954, BD Transduction Laboratories, San Jose, CA, USA), TRPC3 (1:1000, polyclonal, #ab51560, Abcam), TRPC6 (1:1000, polyclonal, #ab62461, Abcam), total Akt (1:2000, monoclonal, #4685, Cell Signaling Technology, Danvers, MA, USA), phosphorylated Akt (1:3000, monoclonal, #4060, Cell Signaling, Danvers, MA, USA), total mTOR (1:1000, polyclonal, #2972, Cell Signaling, Danvers, MA, USA), phosphorylated mTOR (1:1000, polyclonal, #2971, Cell Signaling), total GSK-3β (1:1000, monoclonal, #9315, Cell Signaling, Danvers, MA, USA), phosphorylated GSK-3β (1:1000, polyclonal, #9336, Cell Signaling, Danvers, MA, USA), total β-catenin (1:1000, polyclonal, #ab6302, Abcam), and phosphorylated β-catenin (1:1000, polyclonal, #9561, Cell Signaling, Danvers, MA, USA). The blots were then incubated with secondary antibodies conjugated with horseradish peroxidase. Bound antibodies were detected with the ECL detection system (Millipore, Darmstadt, Germany) and analyzed using AlphaEaseFC software (Alpha Innotech, San Leandro, USA). Targeted bands were normalized to glyceraldehyde 3-phosphate dehydrogenase (GAPDH) protein (1:50,000, monoclonal, #M171-7, MBL, Woburn, MA, USA) to confirm equal protein loading.

### 4.5. Soluble Collagen Measurement

To determine the release of collagen type I, conditioned medium of human cardiac fibroblasts with and without LiCl treatment (1.0 or 0.1 mM)/angiotensin II (50 nM) for 24 h was collected and measured using an enzyme-linked immunosorbent assay (ELISA) kit (#E4617, BioVision Incorporated, Milpitas, CA, USA) following the protocol of the manufacturer.

### 4.6. Transfection of siRNA into Human Cardiac Fibroblast

Human cardiac fibroblasts were transfected with 20 nM of either Orai1 siRNA (#s39560, Thermo Fisher Scientific, Loughborough, UK) or negative control siRNA (#4390843, Thermo Fisher Scientific, Loughborough, UK) using Lipofectamine RNAiMax Transfection Reagent (#13778150, Thermo Fisher Scientific, Loughborough, UK) for 48 h. The protein knockdown efficacy of Orai1 was assessed 48 h after siRNA transfection.

### 4.7. Real-Time Reverse-Transcription Polymerase Chain Reaction (RT-PCR) Analysis

Total RNAs isolated from human cardiac fibroblasts were reversely transcribed using SuperScript III reverse transcriptase (Invitrogen, Carlsbad, CA, USA). Expression levels of Orai1 and GAPDH mRNA were analyzed with a quantitative (q)PCR using the ABI PRISM7300 system (Applied Biosystems, Foster City, CA, USA) and SYBER Green (Applied Biosystems). Relative changes in the transcript levels of Orai1 were estimated from the threshold cycle (Ct) value and normalized against the respective Ct value of the GAPDH reference gene in the corresponding sample. The following primers were used: *ORAI1* (NM_032790): R: 5′-ACTCCTTGACCGAGTTGAGATTG-3′, F: 5′-CTGCATCCTGCCCAACATC-3′; *GAPDH* (NM_002046): R: 5′-CCTGCTTCACCACCTTCTTGA-3′, F: 5′-GGACCTGACCTGCCGTCTAG-3′.

### 4.8. Calcium Fluorescence Imaging

Human cardiac fibroblasts were plated in a 96-well culture dish at a density of 10^4^ cells per well. After growth for 24 h, the cells were incubated in serum-free medium with or without LiCl treatment (1.0 or 0.1 mM) or for 24 h. For the experiment in the human cardiac fibroblasts stimulated with a pro-fibrotic stimulus, representing a pathophysiologically relevant model, angiotensin II (50 nM) was administrated to human cardiac fibroblasts with or without LiCl treatment for 24 h. The cells were then loaded with Fura-2 AM (5 μM; #F1221, Thermo Fisher Scientific) for 30 min at 37 °C. After being washed with Ca^2+^-free phosphate-buffered saline (PBS), the cells were kept in Ca^2+^-free Tyrode’s solution containing NaCl 120 mM, KCl 5.4 mM, KH_2_PO_4_ 1.2 mM, MgSO_4_ 1.2 mM, glucose 10 mM, HEPES 6 mM, and Taurine 8 mM (pH 7.40). Fura-2 fluorescence images were obtained using a Leica DM IL LED microscope (Leica Microsystems, Buffalo Grove, IL, USA) and Andor’s Zyla 4.2 sCMOS camera (Oxford Instruments, Abingdon, Oxfordshire, UK) with the dual-excitation wavelengths of 340 and 380 nm. The exposure time was 50 milliseconds and the measurement interval was 2000 milliseconds. The Fura-2 images were analyzed using MetaFluor software, version 7.7.9.0 (Molecular Devices, Sunnyvale, CA, USA). The amount of cytosolic Ca^2+^ was estimated using the 340/380 nm ratio.

To eliminate the Ca^2+^ in the ER, a sarco/ER Ca^2+^-ATPase inhibitor thapsigargin (2.5 μM; #T9033, Sigma-Aldrich, St. Louis, MO, USA) was added to the cells after baseline 340/380 nm measurement in Ca^2+^-free Tyrode’s solution. After extracellular Ca^2+^ 2 mM was added to cells, the 340/380 nm ratio was measured to assess the degree of store-operated Ca^2+^ entry induced by ER Ca^2+^ depletion. For each independent experiment, we randomly selected 5–10 cells to analyze the maximum amplitude and the AUC of fluorescence intensities.

### 4.9. Immunofluorescence Microscopic Imaging

Human cardiac fibroblasts were plated and cultured in a glass coverslip at a density of 10^4^ cells per well. After growth for 24 h, the cells treated with or without LiCl (0.1 mM)/angiotensin II (50 nM) for 24 h were fixed with 4% paraformaldehyde in PBS for 20 min and permeabilized with 0.1% Triton™ X-100 (#T8787, Sigma-Aldrich, St. Louis, MO, USA) for 10 min. Following being blocked with PBS containing 1% BSA for 1 h, the cells were incubated with primary antibody against α-smooth muscle actin (1:50, monoclonal, #ab7817, Abcam, Cambridge, UK) for 1 h at room temperature. The coverslips were washed three times for 5 min in blocking buffer and incubated for 1 h in the dark using the secondary antibody green anti-mouse IgG (H+L) DyLight^®^ 488 at a dilution of 1:100 (polyclonal, # A90-116D2, Bethyl Laboratories, Montgomery, TX, USA) for α-smooth muscle actin. After incubation in DAPI (300 nM; #D8417, Sigma-Aldrich, St. Louis, MO, USA) for 10 min, the coverslips were washed thrice for 5 min in blocking buffer and subsequently photographed using an Invitrogen EVOS FL Color Imaging System (Thermo Fisher Scientific, Loughborough, UK).

### 4.10. Statistical Analysis

Data are presented in terms of mean ± standard error of the mean. An unpaired *t* test, Mann–Whitney rank-sum test, and one-way analysis of variance (ANOVA) repeated or non-repeated measures) test with Tukey’s post hoc test were used to compare the human cardiac fibroblasts under different treatment conditions. *p* values < 0.05 indicated statistical significance.

## Data Availability

The data presented in this study are available on request from the corresponding author.

## Supplementary Materials

The following are available online at https://www.mdpi.com/1422-0067/22/2/842/s1, Figure S1.
