# Supplementary material for: Lithium Reduces Migration and Collagen Synthesis Activity in Human Cardiac Fibroblasts by Inhibiting Store-Operated Ca2+ Entry
Source: Int J Mol Sci. 2021 Jan 15;22(2):842. doi: 10.3390/ijms22020842 (PMC7830715; doi:10.3390/ijms22020842)
Supplement: Supplementary file 1 [file ijms-22-00842-s001.pdf]

Supplementary Material
Figure S1

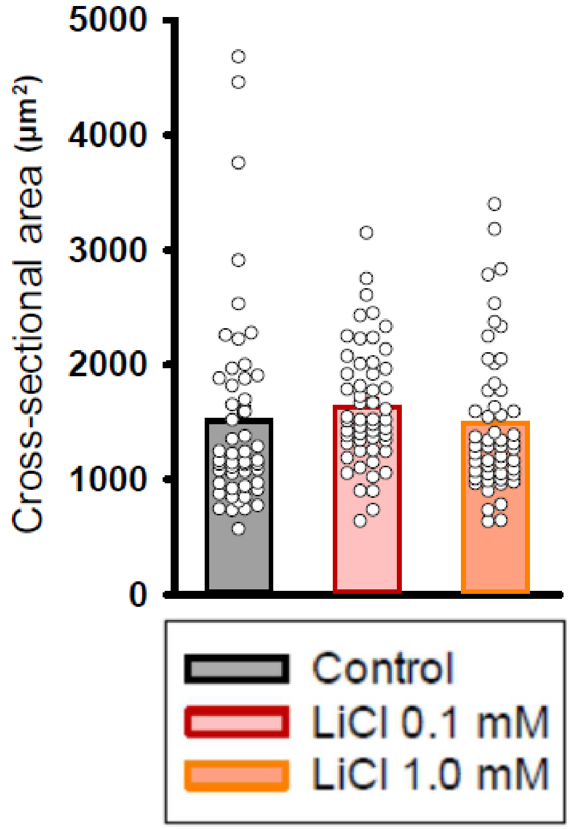

Figure S1. Cell size in human cardiac fibroblasts with and without LiCl treatment for 24 hours. Average data of the cross-sectional area in control (n = 50 cells), LiCl (0.1 mM, n = 54 cells), or LiCl (1.0 mM, n = 52 cells)-treated human cardiac fibroblasts. Cross-sectional areas of cells were analyzed from randomly selected fields to assess the cell size with the Image J. One-way analysis of variance (ANOVA) test with Tukey's post-hoc test was used to compare the human cardiac fibroblasts under different treatment conditions.
